# Mucin Depleted Foci, Colonic Preneoplastic Lesions Lacking Muc2, Show Up-Regulation of *Tlr2* but Not Bacterial Infiltration

**DOI:** 10.1371/journal.pone.0029918

**Published:** 2012-01-05

**Authors:** Angelo Pietro Femia, Alexander Swidsinski, Piero Dolara, Maddalena Salvadori, Amedeo Amedei, Giovanna Caderni

**Affiliations:** 1 Department of Pharmacology, University of Florence, Florence, Italy; 2 Laboratory for Molecular Genetics, Polymicrobial Infections and Bacterial Biofilms, and Section of Gastroenterology, Department of Medicine, Charité Universitätsmedizin Berlin, Berlin, Germany; 3 Department of Internal Medicine, University of Florence, Florence, Italy; 4 Department of Biomedicine, Azienda Ospedaliera Universitaria Careggi (AOUC), Florence, Italy; Lovelace Respiratory Research Institute, United States of America

## Abstract

Mucin depleted foci (MDF) are precancerous lesions of the colon in carcinogen-treated rodents and humans at high risk. Since MDF show signs of inflammation we hypothesized that the defective mucous production would expose them to the risk of being penetrated by intestinal bacteria, which can be sensed by Toll-like receptors (Tlrs) and activate inflammatory pathways. To verify this hypothesis we tested the expression of 84 genes coding for Tlrs and associated pathways using RT-qPCR in MDF (n = 7) from 1,2-dimethylhydrazine (DMH)-treated rats. Among the 84 tested genes, 26 were differentially expressed in MDF with 5 genes significantly up-regulated and 21 down-regulated when compared to the normal mucosa. *Tlr2*, as well as other downstream genes (*Map4k4, Hspd1, Irak1, Ube2n*), was significantly up-regulated. Among the genes regulating the NFkB pathway, only *Map4k4* was significantly up-regulated, while 19 genes were not varied and 6 were down-regulated. Tlr2 protein was weakly expressed both in normal mucosa and MDF. To determine whether inflammation observed in MDF could be caused by bacteria contacting or infiltrating crypts, we performed fluorescence in situ hybridization (FISH) experiments with a rRNA universal bacterial probe. None of the 21 MDF tested, showed bacteria inside the crypts, while among the colonic tumors (n = 15), only one had very few bacteria on the surface and on the surrounding normal mucosa. In conclusion, the up-regulation of *Tlr2* in MDF, suggests a link between this receptor and carcinogenesis, possibly related to a defective barrier function of these lesions. The data of FISH experiments do not support the hypothesis that inflammation in MDF and tumors is stimulated by bacterial infiltration.

## Introduction

Mucin depleted foci (MDF) are precancerous lesions of the colon identified in carcinogen-treated rodents [1] and high-risk humans [2]. MDF carry molecular defects proper of colon tumors such as *Apc* and *Ctnnb1* (coding for β-catenin) gene mutations leading to constitutive activation of the Wnt-signalling [3]–[4]. Phenotipically, these lesions have a defective mucin production since they lack Muc2 expression, the main apomucin in the colon, together with Intestinal Trefoil Factor (ITF or TFF3), a marker of goblet cell lineage, which protects intestinal epithelial cells from various insults and contributes to mucosa repair [5]. Recently, we reported that MDF show increased levels of cyclooxygenase-2 (COX-2), inducible nitric oxide synthase (i-NOS), and macrophage infiltration [6], indicating that detectable levels of local inflammation are present in the very early phases of carcinogenesis. Actually, although high-grade inflammation, as observed in inflammatory bowel diseases (IBD), has been associated with an increased colon cancer risk, the role of low-grade inflammation in colon carcinogenesis is not yet clear [7]. Previous studies reported that genetically deficient Muc2 mice (Muc2-/-) exhibit a higher susceptibility to intestinal carcinogenesis as well as various grades of colon inflammation [8]–[9]. Interestingly, while in normal mice the intestinal flora is separated from the colon mucosa by a densely packed layer of mucus [10]–[11], in Muc2-/- mice bacteria are in direct contact with the mucosa and even penetrate the crypts [11], an observation possibly explaining the proneness of this strain to develop intestinal inflammation and, eventually, cancer [11]–[12]. However, although this rodent genetic model proves the principle that Muc2 deficiency causes a certain degree of inflammation in the colon, it does not imply that focal mucin depletion, as observed in the preneoplastic lesions MDF, may trigger and sustain inflammation through a closer contact with luminal bacteria.

Toll-like receptors (Tlrs) are a family of trans-membrane receptors sensing bacteria in the intestine and connected to cellular pathways which lead to the activation of NF-kB signaling [13]. Beside this function, which is important for defense against infections and tolerance to the commensal flora, Tlrs are implicated in tissue repair and regeneration after injury, activating cell programs leading to proliferation and survival [14]–[15]. Studies conducted mostly in genetically manipulated animal models or cell lines, have associated Tlr activation with colon cancer [16], especially in the context of colitis, that is, in which an overt inflammation is induced with chemicals such as dextran sodium sulphate (DSS) [17]–[20]. The role of these receptors in sporadic colon cancer, in which the role of inflammation is not so clear, is less ascertained [21]–[23].

Recently, we showed that *Tlr2, Tlr5* and *CD180* (a Tlr subtype) genes are up-regulated in colonic tumors of 1,2 dimethylhydrazine (DMH)-induced rats [24], an experimental model for sporadic colon carcinogenesis. Although our previous data [24] suggest a role of these receptors also in the development of colon cancer not driven by clear colitis, their involvement in the early phases of carcinogenesis is not known. On these premises, we thought it of interest to study *Tlr* expression in MDF from rats induced with DMH, performing RT-qPCR of several genes associated to this pathway. Moreover, to verify whether the signs of inflammation observed in MDF could be caused, at least partially, by bacteria contacting or infiltrating MDF crypts, we performed fluorescence in situ hybridization (FISH) with a universal bacterial probe in MDF. FISH experiments on colonic tumors were also performed.

## Methods

### Induction and sampling of MDF for RT-qPCR experiments

Male F344 rats (n = 4), were housed according to the European Union Regulations on the Care and Use of Laboratory Animals [25]; approval of the protocol was received by the Italian Ministry of Health (ID approval 141/2008-B). Rats were treated with s.c. injections of 1,2-dimethylhydrazine (DMH) (150 mg/kg bw ×2 times, one week apart) and sacrificed by CO_2_ asphyxiation 15 weeks later. Colons were fixed in cold absolute ethanol for 3 h before a brief staining with Alcian Blue to identify MDF before plucking out as previously described [6]. For each colon, MDF and equivalent amounts of the corresponding normal mucosa from at least 3 different fields were also harvested for comparison.

### RT-qPCR of Tlr pathway in MDF

Total RNA extraction was performed using the PicoPure™ RNA isolation kit (MDS Analytical Technologies) according to the manufacturer's instruction. RNA from each MDF and corresponding normal mucosa from each colon was retro-transcribed using the RT^2^ Nano Preamp cDNA Synthesis kit (SABiosciences, Qiagen) according to the instructions. Then cDNA was pre-amplified using the specific primer mix for the Tlr pathway (SABiosciences, Qiagen). Pre-amplified cDNA for each individual sample (MDF, n = 7 and corresponding normal mucosa, n = 4) was then aliquoted in the 96 well Toll-like receptors PCR array (SABiosciences, Qiagen) containing 84 genes associated to the Tlr pathway and five housekeeping genes used for normalization (*Rplp1, Hprt1, Rpl13a, Ldha* and *Actb*). Genes present in the plate were: **Toll-Like Receptors:** *TLR7_PREDICTED (Tlr7), Cd180, Tlr1, Tlr2, Tlr3, Tlr4, Tlr5, Tlr6, Tlr9*. **Adaptors & TLR Interacting Proteins:** *Btk, Cd14, Hmgb1, Hras, Hspa1a, Hspd1, Ly96 (MD2), Mal, Mapk8ip3 (JIP3), Myd88, Peli1 (Pellino 1), Pglyrp1 (PGRP-S), Ripk2 (RIP2), Rnf138 (Trif), Sarm1, Ticam2, Tollip*. **Effectors:** *Casp8, Fadd, Irak1, Irak2, Map3k7, Nr2c2 (TAK1), Ppara, Prkr, Traf6, Ube2n (Ubc13), Ube2v1 (Uev1A)*. **Downstream Pathways and Target Genes:** NFκB Pathway: *Ccl2 (MCP-1), Chuk (IKK-a), Csf2 (GM-CSF), Csf3 (G-CSF), Ifna1, Ifnb1, Ifng, Ikbkb (IKK-b), Il1a, Il1b, Il1r1, Il2, Il6, Il10, Il12a, Lta (TNF-b), Map3k1 (MEKK1), Map4k4, Nfkb1, Nfkbia (IkBa/mad3), Nfkbib (IkBb), Nfkbil1, Nfrkb, Rel, Rela, Nfkb2, Tnf (TNFa), Tnfrsf1a, Tnip2 (Tnfaip3), Tradd*. JNK/p38 Pathway: *Fos, Jun, Kcnh8 (Elk1), Map2k3 (MKK3), Map2k4 (MKK4), Map3k1 (MEKK1), Mapk8 (JNK1), Mapk9 (JNK2)*. NF/IL6 Pathway: *Cebpb, Clecsf9, Il6ra, Ptgs2. IRF Pathway: Cxcl10 (IP-10), Ifna1, Ifnb, Ifng, Irf1, Irf3, LOC299827 (Tbk1)*. **Regulation of Adaptive Immunity:** *Cd80, Cd86, Ripk2 (RIP2), Traf6*.

RT-qPCR was carried out in an ABI 7900HT instrument (Applied Biosystems) using the following program: 95°C 15 min followed by 40 cycles of 95°C denaturation for 15 s and 60°C for 1 min.

### Quantification of mRNA expression and statistical analysis

Relative quantification of mRNA expression was carried out using the Delta Delta Ct (2^−ΔΔCT^) method [26]. In detail, for each gene present in the plate, the relative expression (fold change) of each MDF compared to its paired normal mucosa was calculated as 2^−ΔΔCT^, where ΔΔCT is the difference between the ΔCt (Ct of the gene – average Ct of the five housekeeping genes present in the plate) of the MDF and the ΔCt of the corresponding normal mucosa. An average fold change for all the MDF analysed (n = 7) was then calculated.

For each gene present in the plate, the statistical significance of the difference between the expression of MDF and normal mucosa was analysed comparing the ΔCt of the MDF with those of the corresponding normal mucosa using a t-test for paired samples (n = 7). *P* values<0.05 were considered significant.

### Immunohistochemistry of Tlr2 in MDF and normal mucosa

**Figure 1:**
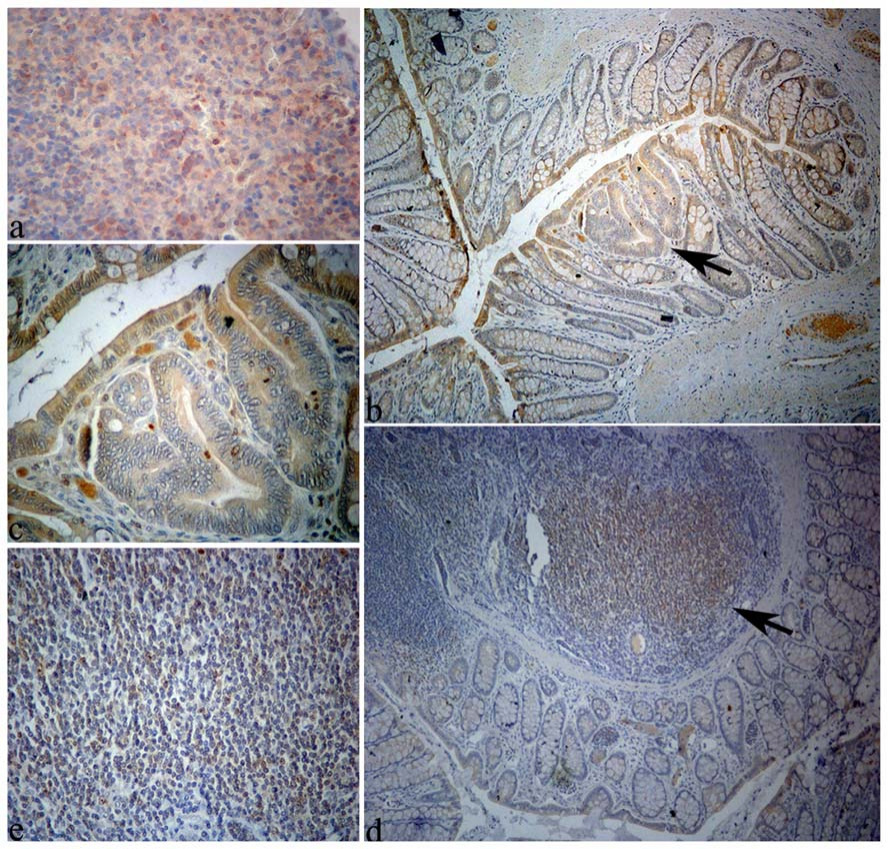
Rat tissues processed with immunohistochemistry to highlight the expression of Tlr2. Panel a: spleen section used as positive control (original magnification, 40×); panel b: colon section containing an MDF (arrow) surrounded by normal mucosa; Tlr2 was weakly expressed by epithelial cells facing the gut lumen in both normal mucosa and MDF (original magnification 10×); panel c: the same MDF of panel c, shown at higher magnification (40×); panel d: colon section with normal mucosa and gut associated lymphatic tissue (GALT, indicated by an arrow) (original magnification 10×): panel e: the same GALT of panel d, shown at higher magnification (40×).

Tlr2 expression was evaluated with immunohistochemistry experiments carried out in longitudinal sections containing both MDF and normal mucosa as previously described [3], [5]–[6]. We used the Tlr2 Antibody Rabbit Polyclonal (Abbiotec, LCC, DanDiego, CA) diluted 1: 100 in PBS and incubated 2 h at room temperature. Positive controls consisting of sections of rat spleen were also included (Fig. 1, panel a).

#### Induction and sampling of MDF for FISH experiments

In a first set of experiments, rats (n = 8) were treated with s.c. injections of DMH (150 mg/kg bw ×2 times) and sacrificed 15 (n = 4) or 23 weeks (n = 4) after the first injection of DMH, to harvest MDF and colonic tumors, respectively. Colons were opened longitudinally without washing, stretched and fixed in cold Carnoy solution (ethanol: glacial acetic acid: chloroform; 6:3:1) for at least 3 hours. Colons were then stained with Alcian Blue-Neutral Red (AB-NR) to identify MDF [4]. Fragments containing MDF were then cleared in toluene and embedded in paraffin to be cut on electrostatic slides (5 µm thick) and analyzed by FISH as described below. In the case of tumors, since these were visible at naked-eye, the colons were not stained with AB-NR. To avoid as much as possible artifacts due to the opening of the colon, in a second set of experiments (n = 8 rats) colons were not opened at sacrifice but immediately fixed in cold Carnoy solution, divided in different segments from proximal to distal before toluene clearing and paraffin embedding. Since these colons were not opened, identification of MDF was carried out by systematically cutting each fragment at microtome. This procedure is time-consuming since long segments of the colon must be cut to identify the lesions; moreover, since fecal pellets within the colon hamper a good sectioning of the mucosa, only empty segments were available for MDF identification. MDF were recognized at histology as focal lesions with absence of goblet cells, distorted crypts and then confirmed by HID-AB staining [1]. In this second set of experiments, to increase the yield of lesions to be harvested, rats were treated with 3 injections of DMH (150 mg/kg for each dose).

### FISH experiments in MDF and tumors

FISH experiments were carried out as previously described [11]: slides with MDF or tumors sections (5 µm thick) were deparaffined and washed in 95% ethanol and then incubated at 50°C with hybridization buffer (20 mM Tris-HCl pH 7.4, 0.9 M NaCl, 0.1% SDS) containing EUB338 probe, a universal bacterial probe (sequence: 5′-GCTGCCTCCCGTAGGAGT-3′) conjugated with FITC (final concentration of the probe: 5 ng/µl, incubation over night) or with the more sensitive fluorocrome CY3 (final concentration of the probe 0.5 ng/µl, incubation 90 min). The sections were then rinsed in wash buffer (20 mM Tris-HCl pH 7.4, 0.9 M NaCl) at 50°C for 20 min, counterstained with DAPI (Vectastain, Burlingame, CA) and observed with a fluorescence microscope (Leica, DM 1000 or Nikon Eclipse 80i) [10], [27].

## Results

### Tlr pathway expression in MDF

The expression of 84 genes coding for Tlrs and associated pathways was studied by RT-qPCR with commercial arrays in MDF (n = 7) and their corresponding normal mucosa. The results showed that among the 84 genes present in the plate, 26 were differentially expressed in MDF (p<0.05) (Table 1), with 5 genes significantly up-regulated and 21 down-regulated when compared to the normal mucosa. *Tlr2* was among the significantly up-regulated genes (Table 1), as well as other downstream genes (*Map4k4, Hspd1, Irak1, Ube2n*). Among the 30 genes classified in the plate as regulators of the NFkB pathway (see the Method section), only *Map4k4* was significantly up-regulated in MDF, while 19 genes were not varied and 6 were down-regulated. Genes coding for *Tlr3, 6* and *7* were significantly down-regulated in MDF. JNK/p38, NF/IL6 and IRF pathways were not significantly affected.

**Table 1:** Genes associated to the Tlr pathway: list of the statistically significant up and down-regulated genes in the MDF.

Gene^a^	Fold change MDF/NM^b^ (Mean±SE)	Gene Function
***Tlr7***	0,4±0,1	Toll-like receptors
***Tlr2***	2,9±0,4	Toll-like receptors
***Tlr3***	0,6±0,1	Toll-like receptors
***Tlr6***	0,6±0,1	Toll-like receptors
***Btk***	0,5±0,2	Adaptors and TLR interacting Proteins
***Hspd1***	1,7±0,1	Adaptors and TLR interacting Proteins
***Ly96***	0,6±0,2	Adaptors and TLR interacting Proteins
***Peli1***	0,6±0,1	Adaptors and TLR interacting Proteins
***Ripk2***	0,7±0,1	Adaptors and TLR interacting Proteins
***Irak1***	1,4±0,2	Effectors
***Ppara***	0,7±0,1	Effectors
***Ube2n***	1,6±0,2	Effectors
***Eif2ak2***	0,5±0,1	Effectors
***Ccl2***	0,5±0,2	NF-KB pathway target genes
***Csf2***	0,5±0,2	NF-KB pathway target genes
***Ifng***	0,1±0,04	NF-KB pathway target genes
***Il10***	0,4±0,1	NF-KB pathway target genes
***Il12a***	0,2±0,1	NF-KB pathway target genes
***Il6***	0,5±0,1	NF-KB pathway target genes
***Map4k4***	1,7±0,4	NF-KB pathway target genes
***Mapk9***	0,7±0,1	JNK/p38pathway target genes
***Irf1***	0,5±0,1	IRF1pathway target genes
***Tbk1***	0,7±0,1	IRF1pathway target genes
***Il6ra***	0,4±0,1	NF/IL6 pathway target genes
***Cd86***	0,5±0,1	Regulation of adaptive Immunity
***Traf6***	0,7±0,1	Regulation of adaptive Immunity

a Genes for which the comparison between the ΔCt of the MDF with that of the corresponding normal mucosa was statistically significant (p<0.05, using t-test for paired samples).

b For each gene, fold change between MDF and normal mucosa was calculated with the 2^−ΔΔCT^ method [26]; values are means±SE (n: 7 MDF analysed).

Given the up-regulation of *Tlr2* gene, immunohistochemistry experiments were carried out to evaluate the expression of its protein in histological sections containing both MDF and normal mucosa (n = 11). Tlr2 was weakly expressed by epithelial cells facing the gut lumen in both normal mucosa and MDF (Fig. 1, panels b and c), however, no differences in Tlr2 expression were observed between MDF and normal mucosa. Positive cells in the lamina propria or within the gut associated lymphatic tissue were also present (Fig. 1, panels d and e).

### FISH experiments

**Figure 2:**
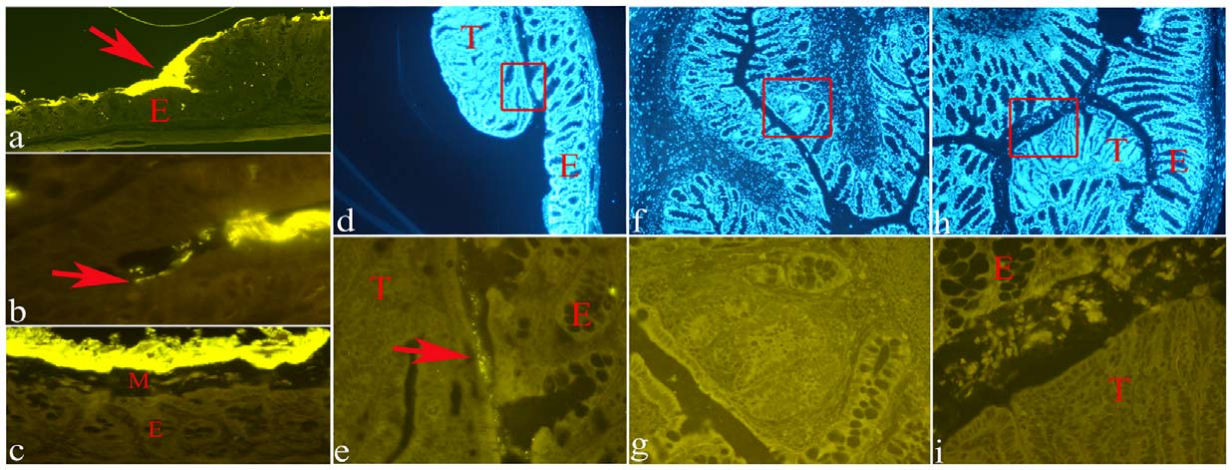
Representative examples of histological sections of rat colon processed with FISH (Cy3-conjugated EUB338 probe). Panel a: normal proximal colon showing a direct contact between bacteria (bright yellow signal) and the intestinal epithelium (E) (original magnification 4×). Panel b: bacteria inside a crypt in the proximal colon (original magnification 100×). Panel c: normal distal colon showing bacteria (bright yellow signal) separated from the epithelium (E) by a layer of mucous (M) (original magnification 40×). Panel d: section of a colonic tumor (T) and its adjacent normal mucosa (E) stained with DAPI (original magnification 4×); the boxed region is shown enlarged in panel e. Panel e: presence of bacteria (arrow) at the interface between tumor (T) and normal mucosa (E) (original magnification 40×). Panel f: section of an unopened colon containing an MDF (boxed) stained with DAPI (original magnification 4×); the boxed region is shown enlarged in panel g. Panel g: no bacteria are present in the MDF (original magnification 40×). Panel h: section of an unopened colon containing a tumor (T) stained with DAPI (original magnification 4×); the boxed region is shown enlarged in panel i. Panel i: no bacteria are present in the tumor (original magnification 40×).

A first set of experiments was carried out with samples (MDF with adjacent normal mucosa) obtained from colon which were opened at sacrifice and then stained with AB-NR, a necessary step to easily and rapidly identify MDF in unsectionned colon. Positive controls consisted of proximal colon in which a direct contact between the mucosa and bacteria has been reported [10]. Accordingly, in this part of the colon, even after staining with AB-NR, we were able to observe a layer of adherent bacteria over the mucosal surface, and, importantly, even inside the crypts (Fig. 2, panels a and b). On the other hand, in the distal part of the colon, bacteria are mainly restricted to the fecal pellets, and therefore are visible in the lumen only if fecal pellets are present (Fig. 2, panel c). Moreover, in the distal part of the colon, where the majority of MDF develop, no bacteria are seen within normal crypts. Similarly, among the 10 MDF processed with FISH, none showed bacteria inside the crypts or in direct contact with the epithelial cells facing the intestinal lumen. Colonic tumors (n = 5) harvested at later time points had no bacteria inside the crypts, but one tumor had very few bacteria on its luminal surface and on its corresponding normal mucosa (Fig. 2, panels d and e). To rule out the possibility that the opening of the colon and the following AB-NR staining, necessary to easily identify MDF in the whole colon, could wash out the few, possible bacteria associated with MDF, we performed a second set of experiments in which MDF were identified histologically with serial sectioning of unopened-unstained colons (see Method sections). This procedure is time-consuming since long segments of the colon must be cut to identify the lesions, moreover, since fecal pellets within the colon hamper a good sectioning of the mucosa, only empty segments were available for MDF identification. Notwithstanding this technical limitation we obtained a good yield of both MDF (n = 11) and tumors (n = 10), that were processed with FISH. Similar to the normal mucosa, no bacteria were present in MDF or in the tumors (Fig. 2, panels f, g, h and i).

## Discussion

The aim of this study was to understand whether the increased expression of inflammatory markers observed in MDF [6] could be related to variations in the expression of *Tlr* and pathways related to these receptors and/or to a direct contact of bacteria with these lesions.

Tlrs are a family of transmembrane receptors sensing conserved molecular patterns associated with bacteria and thus involved in the innate immune response [13]. Tlrs signal through association with proteins like MyD88 finally leading to activation of the NF-kB transcription factor [13]–[15]. In addition to drive inflammatory responses, Tlrs also regulate cell proliferation and survival [14], also through activation of cellular pathways different from NF-kB such as PI3K/Akt or Erk [15], [28]–[29]. Their role in cell proliferation and survival explains their involvement in processes like carcinogenesis and restitution of the colon mucosa after colitis damage [15], [30]. Regarding carcinogenesis in association with colitis, some studies indicated that Tlrs may have a protective role [19]–[20], but other studies suggested the reverse. Accordingly, it has been reported that *Tlr4*-deficient mice are less prone to colon carcinogenesis induced by azoxymethane in association with DSS, an experimental model which mimics colitis-associated carcinogenesis [17]–[18]. Regarding colon carcinogenesis not driven by colitis, it has been reported that the genetic ablation of MyD88, protects mice from colon carcinogenesis, thus suggesting that Tlrs may favor carcinogenesis [16]. We also recently showed that *Tlr2* is over-expressed in DMH-induced colonic tumors [24]. Therefore, the up-regulation of *Tlr2* in MDF observed in this paper is in line with these previous results and suggests an association of this receptor with the early phases of carcinogenesis, a phenomenon which to the best of our knowledge, has not been reported before. However, while the promoting role of Tlrs has been linked to NF-kB signalling, this pathway was not significantly affected in our study, suggesting that the up-regulation of *Tlr2* alone is not sufficient to activate NF-kB. Accordingly, a low activation of NF-kB has been reported in *Tlr2* expressing intestinal cells stimulated by specific ligands, while responsiveness is restored by *Tlr2* transgenic expression [31]. As a matter of fact, despite the up-regulation of the *Tlr2* gene in MDF, we found that its protein was only weakly expressed in MDF as well in normal mucosa. Accordingly, although *Tlr2* mRNA expression in the colon has been documented, the expression of the protein is less ascertained [31]–[33]. Therefore, it is also possible that, despite the increase in *Tlr2* mRNA, the absolute level of the protein is too low to allow the detection of a differential expression with immunohistochemistry.

An other aspect interesting to consider regards the role of Tlrs and Tlr2 in the defence mechanisms that maintain functional tight junctions in the intestinal epithelium [29]. In fact, Tlr2 has been mostly studied in experimental models of overt colitis where it has been reported that Tlr2 regulates the synthesis of TFF3, a main component of the intestinal mucus, contributing to the protection of the intestinal mucosa and to its restitution after damage [34]. Interestingly, we previously showed that MDF and tumors have a defective production of TFF3 (ITF), as well as a decreased expression of its gene [5], [24]. Therefore, it is possible to speculate that a defective production of TFF3, might up-regulate *Tlr2* expression in the attempt to re-establish an efficient layer of mucous.

Regarding bacteria, we found that both MDF and tumors do not show bacterial infiltration nor are in contact with bacteria present in the lumen. The colon is a biofermenter in which bacteria are used to utilize waste products of digestion, recycle water and electrolytes and win additional energy. Bacteria reach concentrations of up to 10^12^ bacteria per gram of feces. The promotion of bacterial growth leads to high diversity of more than 5 000 bacterial species including Bacteroides, E.coli, Enterococci, Clostridium perfringens groups. We regard these species as nonpathogenic, since they can be found in each healthy colon. This assumption is however false. The “nonpathogenity” of many colonic bacteria is not intrinsic, but due to intact mechanisms of the host protection. A defect in protection can lead to deadly diseases caused by the same bacteria, such as abscesses, sepsis, endocarditis or gas gangrene. One of protective mechanisms in the colon is a separation of the biofermentative compartment from the mucosa by an impenetrable mucus layer. As long as the separation of pathogens is perfect, the concentration of bacteria within the large intestine is nonproblematic and may remain high. Accordingly, a mucus gel covering the colonic mucosa and separating the luminal bacteria from the epithelial surface through the colon has been demonstrated in both humans and experimental animals [10], [27], [35]. Alterations in this mucous layer, as observed in Muc2 -/- mouse colon allows bacteria to have a direct contact with epithelium, and, based on this result, it has been hypothesized that the close contact would trigger inflammation and possibly carcinogenesis [11]. This hypothesis is attractive, since, although many studies established that the intestinal microflora affects carcinogenesis with various mechanisms (e.g. production of beneficial or harmful metabolites), it is not clear whether bacteria affect colon carcinogenesis also through a direct contact with the intestinal mucosa. Regarding our results, we observed, as previously reported by others [35], that in distal part of the normal colon, bacteria are mainly restricted to the fecal pellets and therefore are visible in the lumen only if fecal pellets are present. Due to technical limitations, we could not study MDF in association with fecal pellets, and therefore, we can not exclude that a transient, direct contact between MDF and bacteria takes place at the passage of fecal pellets. Certainly, our results showed that, despite their defective mucous production, bacteria do not infiltrate MDF or tumors. One possible explanation for this result is that in both MDF and tumors, the residual mucous production (mostly Muc5ac [5]), may be sufficient to protect these lesions. It is also possible that the mucous produced by normal adjacent cells (mostly Muc2), may compensate the deficiency of mucus production in MDF. However, this seems not be the case for tumors, whose surface, protruding into the intestinal lumen (see Fig. 2, panel h) would be too large to be protected by the mucous of adjacent cells. Another possibility to explain the lack of bacteria in MDF and tumors, could be related to the overexpression of antimicrobial α-defensins, that we recently reported in DMH-induced tumors [2]. Accordingly, hyperproduction of α-defensins has been recently related to a lower number of bacteria adhering to adenomatous polyps [36], a mechanism that could be plausible also for MDF, since defensin production is controlled by Wnt signaling, constitutively activated in MDF [3].

In conclusion, despite a supposed role of Tlr-activated pathways in colon carcinogenesis, the present results do not show a significant Tlr-induced NF-kB activation in MDF. However, the up-regulation of *Tlr2* in MDF as well in tumors, suggests a link between this receptor and carcinogenesis, that could be related, at least in part to defective barrier function of these lesions. Moreover, the data of FISH experiments do not support our hypothesis that inflammation in MDF is stimulated by bacterial infiltration.
